# Simplified sigmoidal curve fitting for a 6 MV FFF photon beam of the Halcyon to determine the field size for beam commissioning and quality assurance

**DOI:** 10.1186/s13014-020-01709-x

**Published:** 2020-12-07

**Authors:** Min-Geon Choi, Martin Law, Do-Kun Yoon, Mikoto Tamura, Kenji Matsumoto, Masakazu Otsuka, Moo-Sub Kim, Shih-Kien Djeng, Hajime Monzen, Tae Suk Suh

**Affiliations:** 1 Department of Biomedical Engineering and Research Institute of Biomedical Engineering, College of Medicine, The Catholic University of Korea, Seoul, 06591 Republic of Korea; 2 Department of Medical Physics, Graduate School of Medical Sciences, Kindai University, Osaka-Sayama-Shi, 377-2, Ohno-Higashi, Osaka-Sayama-Shi, Osaka, 589-8511 Japan; 3 Department of Radiology, Kindai University Hospital, Osaka-Sayama-Shi, 377-2, Ono-Higashi, Osaka-Sayama-Shi, Osaka, 589-8511 Japan; 4 Proton Therapy Pte Ltd., 1 Biopolis Drive, Singapore, 138622 Singapore

**Keywords:** Field size, Sigmoidal curve fitting, FFF, Halcyon, Commissioning, QA

## Abstract

**Background:**

An O-ring gantry-type linear accelerator (LINAC) with a 6-MV flattening filter-free (FFF) photon beam, Halcyon, includes a reference beam that contains representative information such as the percent depth dose, profile and output factor for commissioning and quality assurance. However, because it does not provide information about the field size, we proposed a method to determine all field sizes according to all depths for radiation therapy using simplified sigmoidal curve fitting (SCF).

**Methods:**

After mathematical definition of the SCF using four coefficients, the defined curves were fitted to both the reference data (RD) and the measured data (MD). For good agreement between the fitting curve and the profiles in each data set, the field sizes were determined by identifying the maximum point along the third derivative of the fitting curve. The curve fitting included the field sizes for beam profiles of 2 × 2, 4 × 4, 6 × 6, 8 × 8, 10 × 10, 20 × 20 and 28 × 28 cm^2^ as a function of depth (at 1.3, 5, 10 and 20 cm). The field size results from the RD were compared with the results from the MD using the same condition.

**Results:**

All fitting curves show goodness of fit, R^2^, values that are greater than 0.99. The differences in field size between the RD and the MD were within the range of 0 to 0.2 cm. The smallest difference in the field sizes at a depth of 10 cm, which is a surface-to-axis distance, was reported.

**Conclusion:**

Application of the SCF method has been proven to accurately capture the field size of the preconfigured RD and the measured FFF photon beam data for the Halcyon system. The current work can be useful for beam commissioning as a countercheck methodology to determine the field size from RD in the treatment planning system of a newly installed Halcyon system and for routine quality assurance to ascertain the correctness of field sizes for clinical use of the Halcyon system.

## Background

The Halcyon (Varian Medical Systems Inc., Palo Alto, USA), which is a linear accelerator (LINAC) with an O-ring gantry, is a radiotherapy machine with a 6-MV flattening filter free (FFF) beam, such as the Tomotherapy and Cyberknife systems. In the case of the Halcyon, because the commissioning and quality assurance (QA) processes are totally different than those of the conventional LINAC process, an approach has been developed for the users. The conventional LINAC process required strict and long-time measurements for the commissioning and QA steps, while the Halcyon system provides an independent opportunity to verify the consistency of the measured data (MD) during the commissioning and QA processes with the reference data (RD) provided by the vendor. The RD includes parameters such as the percentage depth dose (PDD), dose profile, and output factor, and the new commissioning process of the Halcyon generally follows the guidelines of Association of Physicists in Medicine (AAPM) MMPG 5.a, AAPM TG-51 and TG-100 to satisfy international commissioning standards [1–3]. Therefore, many studies have checked and verified various parameters other than the basic parameters for the commissioning and QA processes of the Halcyon, and these parameters could be applied as another factor to verify the accuracy of the commissioning and QA processes of the Halcyon [4, 5]. The purpose of this study is to demonstrate the method for determining the field size of the Halcyon system using the simplified sigmoidal curve and to provide a field size parameter dataset that can improve the effectiveness of the commissioning and QA processes.

In radiotherapy, when using a LINAC, the field size of the radiation beam refers to the area of radiation delivery. For this reason, determination of accurate field sizes is a significant parameter for the delivery of radiation and an important process in commissioning and quality assurance (QA) [6, 7]. Normally, the field size can be determined during a commissioning and QA procedure [8, 9]. While conventional LINAC systems are often equipped with a flattening filter (FF) to deliver the radiation beam with a uniform dose distribution, the full width at half maximum (FWHM) method is the conventional representative methodology for determining the field size of the FF beam [10]. The field size of the FF beam is defined based on a point off-axis at a dose of 50% after dose normalization of a central axis (CAX) at 100%. This FWHM methodology is suitable for determination of the field size of the FF beam because it has a uniform region around the CAX. According to the Task Group Report #142 of AAPM, several parameters, such as flatness, symmetry and penumbra, should be considered when characterizing the FF beam [11]. Currently in radiation therapy, the accuracy of the dose is a very important factor, and the treatment time should not be overlooked. Because a long treatment time may cause patient discomfort and decrease the accuracy of treatment, a flattening filter-free (FFF) beam is used to reduce the treatment time. The FFF beam has the effect of reducing photon head scatter, leaf transmission head leakage and the peripheral dose [12, 13]. However, the FWHM method is not suitable for determining the field size of the FFF beam, which shows a specific shape of the dose profile, with a relatively higher peak at the CAX.

Several studies have defined the field sizes of an FFF beam. The most representative method to determine the field size of an FFF beam is to use the inflection point (IP) on the penumbra region of the beam’s profile [14, 15]. Nevertheless, some uncertainties remain in obtaining a correct IP from beam data measurements. To consider this uncertainty, Pönisch et al. proposed a method to identify the IP at the field edge of an FFF beam with the same level as that of an FF beam. The position of the IP can be changed according to the positional step size error to obtain the beam profile [16]. Fogliata et al. suggested a renormalization formulation to overcome the uncertainty in the IP due to the stepping size [17]. Although these two methods to define the field size of the FFF beam are based on the profiles of the FF beam and thus include a large number of values, they both exhibit a position error during measurement. The parameterized gradient-based method (PGM) that complements these two methods was proposed to determine the field size of the FFF beam using a mathematical model. Although the PGM results were effective for determining the field size, this method did not yield parameters for the specific field size. We struggled to determine the reason why some data were omitted. Some institutes may want the omitted data according to the level of an instrument or the requirements of a specific treatment case [18, 19].

In addition, because the PGM method also applies a mathematical model based on MD, uncertainty still exists in the measurements, as in the initial two methods. When users of the Halcyon use our proposed method to determine the field size, there are several convenient benefits. First, we proposed a reasonable method to determine the field size of the Halcyon beam according to all depths and all field sizes. The vendor of the Halcyon has already entered all beam data into the Eclipse TPS. This RD is used to optimize and calculate the dose, and this approach is effective for operation of the Halcyon in the Eclipse TPS. However, because the vendor does not provide any information regarding the field size in the RD, the field size should be determined by their method for all conditions. In this study, one of the methods for determining the effective value of the field size with a simple mathematical equation was employed for all depths and all field sizes.

Second, we used the RD from the vendor to determine the field size and dose without using our MD. The commissioning process of the Halcyon does not require an adjustment of the beam model by the user in the way that conventional LINAC commissioning does. Instead, the user should check the degree of correspondence between the RD included in the installed TPS and the MD to confirm the criteria of commissioning. This can reduce the possibility of errors induced by unexpected conditions during commissioning and the QA process in the conventional LINAC, and it can provide an opportunity to verify the reliability and accuracy of beam data by comparing the degree of correspondence between the RD and the MD. When we measure the data for the additional commissioning procedure or QA steps, we can check the accuracy of the field size using the reference value from the RD.

Finally, we used the methodology with only the sigmoid function without any other equations to generate the fitting curve on the beam profile. The procedure for the proposed method that is applicable to the RD has been simplified compared with the procedure of the PGM.

## Methods

### Preparation of data

A preconfigured reference beam dataset (RD) generated by the vendor is stored in the treatment planning system when a new Halcyon system is installed. The RD includes the lateral dose profiles for field sizes of 2 × 2, 4 × 4, 6 × 6, 8 × 8, 10 × 10, 20 × 20 and 28 × 28 cm^2^ as a function of depth at 1.3, 5, 10 and 20 cm in the water phantom. To compare the field size from the RD with that from the MD, the measurement was performed under the same conditions as those used to obtain the RD. The source-to-surface distance (SSD) was set at 90 cm. A CC13 ionization chamber and a Blue Phantom water tank (IBA Dosimetry, Schwarzenbruck, Germany) were used to measure the relative dose profiles for field sizes > 4 × 4 cm^2^. For field sizes ≤ 4 × 4 cm^2^, an edge diode detector (Sun Nuclear, Melbourne, FL, USA) was used. The scanning step for acquisition of the profile on the measurement line along the off-axis position was 0.1 cm. All measurement values were processed with OmniPro Accept7 (version 7.4.24.0) software (IBA dosimetry, Schwarzenbruck, Germany).

### Definition of fitting using sigmoidal curve

The sigmoidal curve originates from the sigmoid function, which is used in the field of the signal process. The shape of the sigmoidal curve is given by Eq. (1),

1 $${\varvec{f}}\left({\varvec{x}}\right)={\varvec{\gamma}}\left(\frac{1}{1+{{\varvec{e}}}^{{\varvec{a}}{\varvec{x}}+{\varvec{\beta}}}}\right)+{\varvec{\delta}}$$

The coefficients α, β, γ and δ are used to determine the shape of the curve *f(x)*. The coefficient α controls the gradient of the sigmoidal curve. The higher the value of α is, the steeper the curve gradient. The coefficient β is related to the horizontal movement of the entire sigmoidal curve. The higher the value of β is, the further the sigmoidal curve moves to the right. The coefficient γ determines the location of the only upper end of the sigmoidal curve. The higher the value of γ is, the higher the position of the upper end of the sigmoidal curve. The coefficient δ determines the vertical movement of the entire sigmoidal curve. The higher the value of δ is, the more upward the direction of the sigmoidal curve. Thus, the coefficients α and γ contribute to transforming the shape of the curve. The coefficients β and δ change the location of the sigmoidal curve.

After uploading the profile to MATLAB (2019 version, MathWorks Inc, Sherborn, MA, USA), sigmoidal curve fitting (SCF) was performed by changing each coefficient until the sigmoidal curve overlapped the profile. To fit the sigmoidal curve to the profile, four steps are required. First, the gradient level of the sigmoidal curve should be the same as the gradient of the profile. When these two gradients agree, the coefficient of α is obtained. The second step is to let the sigmoidal curve move so that it overlaps the profile through the horizontal pitch using the coefficient β. In the third step, the coefficient γ is altered to adjust the upper limitation of the sigmoidal curve so that the curve stops at the upper end of the profile. Similarly, in the last step, the coefficient δ is altered to adjust the lower limitation of the sigmoidal curve so that it stops at the lower end of the profile. In this process, we are able to change all the coefficients to edit the shape of the sigmoidal curve fitting. If the fitting curve is normal, an S-shaped curve that exactly overlaps the RD curve will be obtained.

### Verification of agreement for fitting curves

To verify the accuracy of the fitting curve based on the sigmoidal curve with the profiles, the average agreement ratio (AAR) between the values in the fitting curve (f_i_) and the values in the profiles (x_i_) at the same step position was calculated using Eq. (2), which shows the agreement between the profile and fitting values.

2 $${\text{AAR }}\left( \% \right) = 100 - \frac{1}{n}\sum\limits_{i} {\left( {\left( {\frac{{\left| {x_{i} - f_{i} } \right|}}{{x_{i} }}} \right)~ \times ~100} \right)}$$

In this study, if the AAR is higher than 97%, the fitting optimization terminates because a sufficient accuracy has been obtained, and the four coefficients (α, β, γ and δ) are used to define the shape of the final fitting curve. Moreover, an additional verification was performed based on the evaluation of goodness of fit, R^2^ (Eq. 3).

3 $${R}^{2} = 1 - \frac{{\mathop \sum \nolimits_{{i}} ({f}_{{i}} {~} - {y}_{{i}} )^{2} }}{{\mathop \sum \nolimits_{{i}} ({y}_{{i}} {~} - {~\bar{f}})^{2} }}$$

where $\overline{f}$ is the mean of all *f*_*i*_ values on the fitting curve, and *y*_*i*_ is a value on the profile. The same validation procedures were applied to the MD and the RD.

### Identification of specific regions and points

**Fig. 1:**
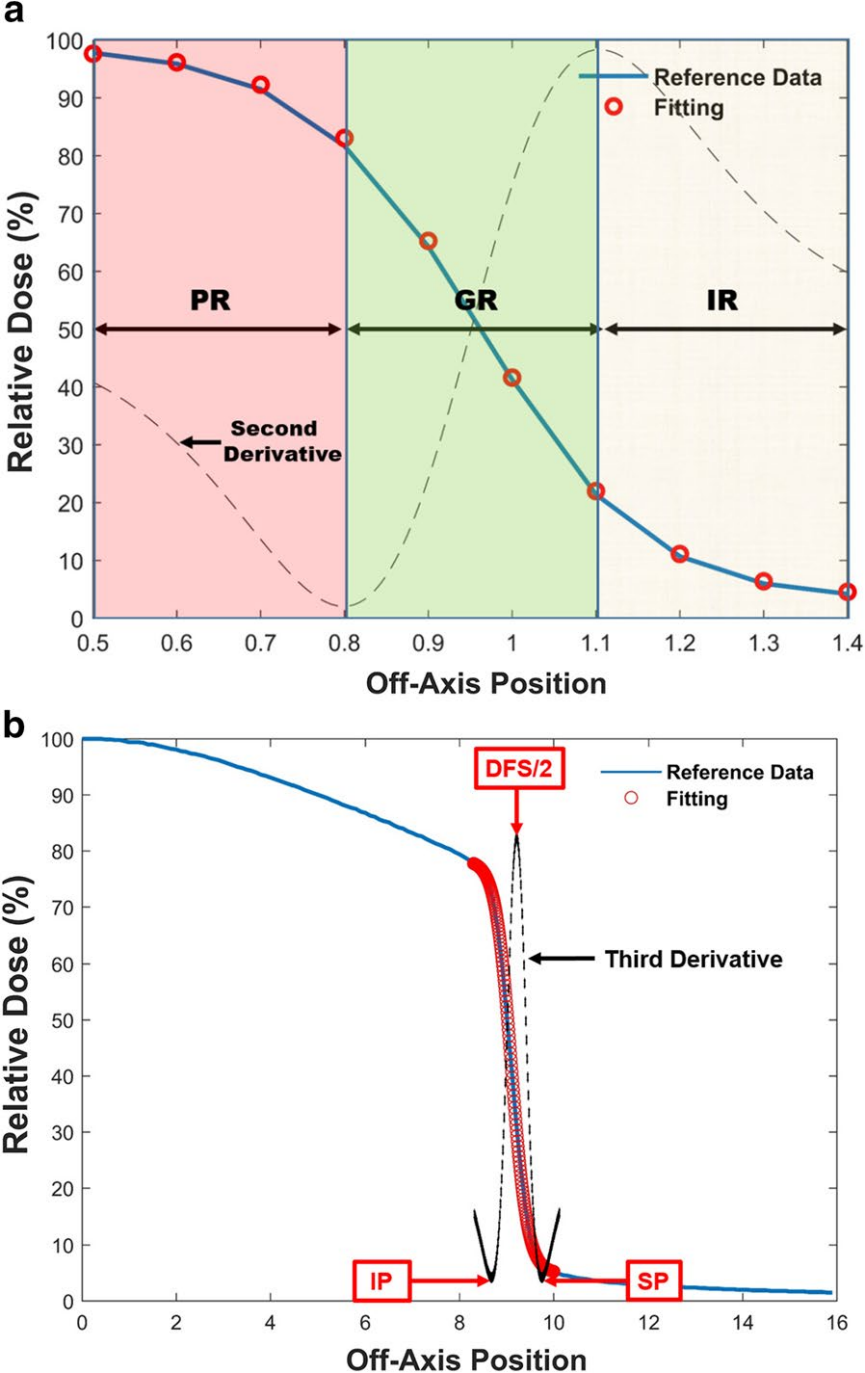
**a** An example to explain the method for identification of three regions and two specific points. The three regions were defined by the second derivative, and the two specific points were set at the minimum points of the third derivative curve: Identification of the position of the introductory region (IR; Yellow region), the growing region (GR; Green region) and the plateau region (PR; Red region). **b** The singular point (SP) and the inflection point (IP). The red dots show the fitting curve on the profile (Blue line). The black dotted line shows the shape of the third derivative curve. A comparison of the entire fitting curve with the third derivative: the positions of the SP, IP and DFS/2 on the black dotted line are shown

In this study, to describe the sigmoidal curve, three regions and two points were assigned in the definition of the half-side of the SCF (Fig. 1a). The three regions include the introductory region (IR), the growing region (GR) and the plateau region (PR). The IR is the region where the sigmoidal curve begins to increase. The GR is a continuously increasing region on the sigmoidal curve. The PR is the region where the increase slows. These regions can be identified through the second derivative of the sigmoidal curve, as shown Fig. 1a. The range between the rightmost point and the maximum point on the second derivative curve is defined as the IR. The range between the maximum point and the minimum point on the second derivative curve is defined as the GR. The region between the minimum point and the leftmost point on the second derivative curve is defined as the PR. Because of the specification of the sigmoidal shape, there are two specific points: the singular point (SP) and the IP, both of which can be identified from the third derivative curve of the sigmoidal curve. The SP is the minimum point between the range of the IR and the GR (Eq. 4). The IP is another minimum point in the range between the GR and the PR, as shown in Eq. (5). When there is no point in either the IP or the SP, the refitting process from Sect. 2.2 is performed.

4 $${\text{SP}}({\text{IR}} < {\text{x}} < {\text{GR}}) = {\text{Min}}\left( {\frac{\partial }{{\partial x}}\frac{\partial }{{\partial x}}\frac{\partial }{{\partial x}}\gamma \left( {\frac{1}{{1 + e^{{\alpha x + \beta }} }}} \right) + \delta } \right)$$

5 $${\text{IP}}({\text{GR }} < {\text{x}} < {\text{PR}}) = {\text{Min}}\left( {\frac{\partial }{{\partial x}}\frac{\partial }{{\partial x}}\frac{\partial }{{\partial x}}\gamma \left( {\frac{1}{{1 + e^{{\alpha x + \beta }} }}} \right) + \delta } \right)$$

#### Determination of the field size

Although the determined field size (DFS) can be calculated using only the first derivative, the second and third derivatives provide an opportunity to check whether or not a given DFS exists in the period between the IP and the SP and to assess the error of the fitting curve. Incorrect fitting or an insufficient fitting range for the first derivative can be defined as the wrong field size. In the extreme case, the IP and the SP cannot be found on the derivative curve. As a result, the DFS cannot be calculated. If we determine the field size using only the first derivative, the results will contain some uncertainties. Therefore, the purpose of the second derivative is to check for the presence of both the IP and the SP. Finally, the purpose of the third derivative is to determine the DFS for the clear periods between the IP and the SP. Because the right third derivative curve can show these three points at the same time, determining the field size using the third derivative curve is the most efficient approach. After the SP and the IP have been obtained, the DFS can be identified as the maximum point on the third derivative curve between the SP and the IP, as shown below in Eq. (6). Figure 1b shows the conceptual DFS on the third derivative curve, and an actual example of the DFS is presented by fitting the profile in Fig. 1b.

6 $${\text{DFS}} = 2*{\text{Max}}\left( {\frac{\delta }{{\delta {\text{x}}}}\frac{\delta }{{\delta x}}\frac{\delta }{{\delta x}}\gamma \left( {\frac{1}{{1 + e^{{(\alpha x\left( {SP < x < IP} \right) + \beta }} }}} \right) + \delta } \right)$$

The factor 2 is used in Eq. (6) because only the right half of the symmetric open beam profile was used for the curve fitting.

## Results

### Accuracy of the fitting curve

**Fig. 2:**
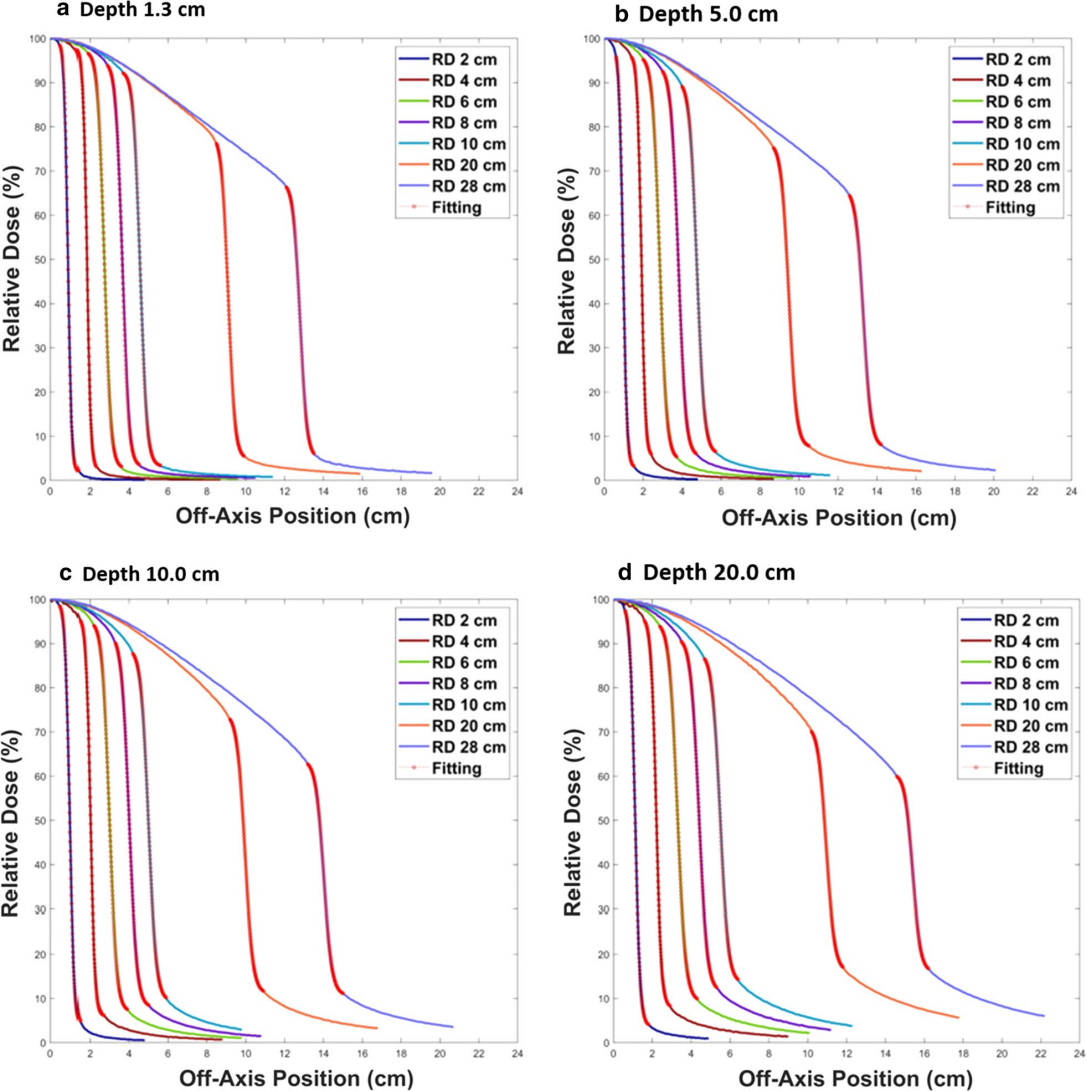
The fitting curves and the profiles from the reference data (RD). The profiles according to the field sizes of 2 × 2, 4 × 4, 6 × 6, 8 × 8, 10 × 10, 20 × 20 and 28 × 28 cm^2^ along with the fitting curves for each beam profile (red circles). The depths for the profiles are **a** 1.3 cm, **b** 5 cm, **c** 10 cm and **d** 20 cm

**Fig. 3:**
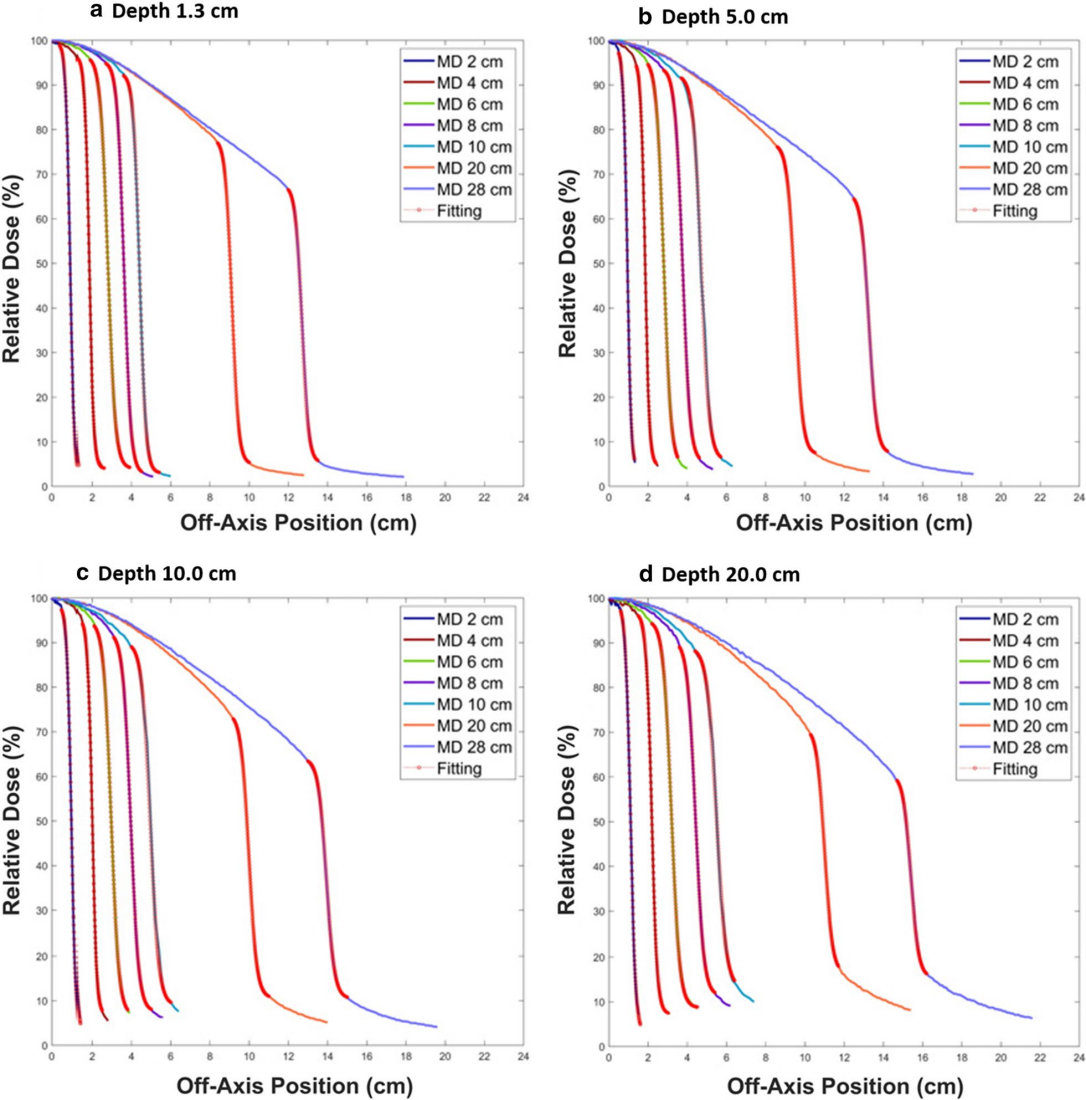
The fitting curves and the profiles from the measured data (MD). The profiles according to the field sizes of 2 × 2, 4 × 4, 6 × 6, 8 × 8, 10 × 10, 20 × 20 and 28 × 28 cm^2^ along with the fitting curves for each beam profile (red circles). The depths for the profiles are **a** 1.3 cm, **b** 5 cm, **c** 10 cm and **d** 20 cm

Figure 2 shows all the final fitting curves using SCF with half of the profiles in the RD. The profiles for all field sizes (2 × 2, 4 × 4, 6 × 6, 8 × 8, 10 × 10, 20 × 20 and 28 × 28 cm^2^) are demonstrated as a function of depth (1.3, 5, 10 and 20 cm) in (a), (b), (c) and (d), respectively. The fitting curve is indicated by the red circles on each profile. In contrast, Fig. 3 shows all the final fitting curves using the same SCF with half of the profiles in the MD. This figure also includes the profiles for all field sizes with variable depths: depths of 1.3, 5, 10 and 20 cm are shown in (a), (b), (c) and (d), respectively. The profiles in Figs. 2 and 3 along the field size are distinguished by their color and have been normalized according to the relative dose at the CAX. The X-axis shows the off-axis position from the CAX. All the fitting curves show good agreement with each profile. The accuracy of all the fitting curves was evaluated using the AAR and R^2^ methods. The R^2^ values were all greater than 0.99.

Table 1 tabulates all coefficients to form the final sigmoidal curves for all field sizes with all depths using SCF before determination of the field size. The differences of the coefficient values at all depths were reported as a maximum difference of α of 1.3, a maximum difference of β of 4.4, a maximum difference of γ of 4.0, a maximum difference of δ of 1.7, and all coefficients had a minimum difference of 0. All IP and SP values were also tabulated. The maximum and minimum IP differences were 0.15 cm and 0 cm, respectively. The maximum and minimum SP differences were 0.18 cm and 0 cm, respectively.

**Table 1:** The coefficients to form the final sigmoidal curves for all profiles and all values for the IP and the SP

	Field Size	RD	MD	RD	MD	RD	MD	RD	MD	RD	MD	RD	MD
	(cm^2^)	α	α	β	β	γ	γ	δ	δ	IP (cm)	IP (cm)	SP (cm)	SP (cm)
	2 × 2	11.20	9.90	− 10.30	− 9.20	97.50	97.50	1.80	2.00	0.69	0.66	1.10	1.14
	4 × 4	10.00	8.80	− 18.30	− 16.20	97.50	93.50	2.30	4.00	1.58	1.56	2.03	2.08
Depth	6 × 6	6.06	5.20	− 16.50	− 14.50	94.50	93.00	2.90	4.00	2.32	2.32	3.08	3.20
1.30 cm	8 × 8	6.03	5.70	− 22.00	− 20.60	92.00	93.00	3.30	2.80	3.24	3.19	4.00	3.99
	10 × 10	6.01	6.30	− 27.50	− 27.80	89.50	89.60	3.20	3.00	4.17	4.02	4.93	4.75
	20 × 20	5.85	5.90	− 53.50	− 53.80	73.00	73.00	4.90	5.00	8.91	9.01	9.78	9.80
	28 × 28	5.70	5.90	− 72.30	− 74.90	62.50	62.50	5.00	5.00	12.37	12.28	13.18	13.06
	2 × 2	10.20	9.80	− 9.80	− 9.20	95.00	95.00	3.50	3.40	0.71	0.68	1.16	1.15
	4 × 4	10.00	9.40	− 19.00	− 17.40	90.50	90.70	5.90	4.70	1.65	1.58	2.10	2.07
Depth	6 × 6	5.50	5.30	− 15.60	− 14.80	91.00	91.00	5.00	4.60	2.39	2.33	3.23	3.20
5.00 cm	8 × 8	5.30	5.30	− 20.10	− 19.90	88.00	88.00	6.00	5.40	3.33	3.30	4.20	4.16
	10 × 10	5.30	4.80	− 25.20	− 24.60	84.30	85.80	6.30	5.90	4.30	4.21	5.12	5.16
	20 × 20	5.10	5.10	− 48.30	− 48.40	70.00	69.60	7.20	7.20	9.00	9.02	9.93	9.91
	28 × 28	5.00	5.00	− 66.50	− 66.30	60.00	59.50	7.00	6.80	12.82	12.78	13.73	13.69
	2 × 2	8.70	8.80	− 8.70	− 8.80	96.00	96.40	3.50	2.10	0.73	0.71	1.22	1.24
	4 × 4	9.00	8.80	− 18.10	− 17.50	90.00	88.50	6.00	6.90	1.73	1.70	2.24	2.22
Depth	6 × 6	5.50	5.10	− 16.40	− 15.10	88.00	88.00	7.30	6.80	2.54	2.49	3.37	3.39
10.00 cm	8 × 8	5.10	4.90	− 20.40	− 19.40	86.50	84.80	7.40	7.60	3.53	3.47	4.42	4.40
	10 × 10	5.10	4.60	− 25.50	− 22.80	81.80	91.00	8.30	9.00	4.53	4.51	5.42	5.44
	20 × 20	4.90	4.90	− 48.80	− 48.90	64.70	63.60	10.60	10.60	9.47	9.49	10.40	10.42
	28 × 28	4.80	4.80	− 67.30	− 66.90	53.50	53.50	10.50	10.50	13.52	13.43	14.47	14.39
	2 × 2	7.80	7.70	− 8.65	− 8.50	94.50	96.20	4.00	2.90	0.79	0.78	1.38	1.38
	4 × 4	7.50	7.10	− 16.50	− 15.50	89.70	89.40	7.30	7.30	1.87	1.84	2.48	2.48
Depth	6 × 6	4.80	4.80	− 15.80	− 15.30	97.00	86.30	8.40	8.70	2.79	2.72	3.74	3.71
20.00 cm	8 × 8	4.80	4.80	− 21.10	− 21.20	82.00	79.20	10.50	11.40	3.89	3.91	4.85	4.87
	10 × 10	4.90	4.10	− 26.90	− 22.50	75.50	76.00	13.50	13.00	5.00	4.90	5.93	6.02
	20 × 20	4.75	5.00	− 54.30	− 55.00	56.00	54.30	16.00	16.40	9.62	9.60	10.45	10.40
	28 × 28	5.00	5.00	− 77.00	− 77.00	45.50	45.00	15.50	15.50	14.22	14.22	14.68	14.70

Reference Data: RD, Measured Data: MD, Singular Point: SP, Inflection Point: IP

Table 2 lists all DFS values from the fitting curve for all the profiles at different depths. For the field sizes, the maximum difference was 0.2 cm, and the minimum difference was 0 cm. The results for a depth of 10 cm showed the least difference between the RD and the MD.

**Table 2:** The determined field sizes from the fitting curves for all profiles

	Field Size (cm^2^)	RD DFS (cm)	MD DFS (cm)
	2 × 2	1.80	1.82
	4 × 4	3.64	3.66
Depth	6 × 6	5.42	5.54
1.30 cm	8 × 8	7.26	7.20
	10 × 10	9.12	9.00
	20 × 20	18.18	18.20
	28 × 28	25.56	25.36
Depth	2 × 2	1.90	1.84
5.00 cm	4 × 4	3.76	3.68
	6 × 6	5.64	5.56
	8 × 8	7.56	7.48
	10 × 10	9.44	9.38
	20 × 20	18.94	18.96
	28 × 28	26.56	26.50
Depth 10.00 cm	2 × 2	1.98	1.96
	4 × 4	4.00	3.94
	6 × 6	5.94	5.90
	8 × 8	7.96	7.88
	10 × 10	9.96	9.98
	20 × 20	19.88	19.92
	28 × 28	28.02	27.96
Depth 20.00 cm	2 × 2	2.18	2.18
	4 × 4	4.38	4.34
	6 × 6	6.56	6.44
	8 × 8	8.76	8.80
	10 × 10	10.94	10.94
	20 × 20	21.68	21.90
	28 × 28	30.76	30.96

Reference Data Determination of Field Size: RD DFS, Measured Data Determination of Field Size: MD DFS

## Discussion

Previous studies have been conducted to determine the field size of the conventional FF beam using methods such as the FWHM, which is not suitable for the LINAC profile in the Halcyon due to the difference in the CAX of the dose. The limitation of the current measurement is that it is time consuming to apply a fine scanning step of 0.1 cm in the QA and commissioning processes. MSM, techniques developed by Pönisch et al. and the method of renormalization, all of which are typical methods for defining the FFF beam field size, can contain uncertainty as a result of the scanning step [14–17]. The uncertainty in the location of the IP may occur from the MD due to the use of a scanning step with a lower resolution. In contrast, although the PGM method has the advantage of not affecting the size of the scanning step, its analytical fitting procedure also obtains the parameters from the MD. In the case of PGM, the field size of the MD is largely separated as 4 × 4 cm^2^ ≤ and 4 × 4 cm^2^ > to obtain the coefficient from the MD. However, the coefficients for 2 × 2 cm^2^ and 2.5 × 2.5 cm^2^ in the calculated data, which are used to check the accuracy of the coefficients of the MD, are not presented. Although this does not rule out the possibility of equipment-specific changes, the field size of the small field is an important factor for equipment from any vendor [18, 19]. Thus, all the approaches described above define the field size based on data measured by the user, whereas our method defines the field size using the RD, which represents the characteristics of the Halcyon. This means that the consistency of the beam data is guaranteed for all institutions using the Halcyon, and the use of mathematical fitting curves to determine Halcyon field size ensures a high accuracy and reliability; further, commissioning and QA can be conducted at each institution based on the RD [2–5]. In our study, the field size of the FFF beam was defined in a simpler and more intuitive way than the PGM method, and the process was simplified while reducing the possibility of errors in the commissioning and QA steps by using the RD. These results are meaningful because a reference criterion is provided to Halcyon users that can be used to compare results with a full dataset. Adequate parameter information and a simple methodology could be useful, especially for new Halcyon users who must validate the preconfigured reference for the beam commissioning process and perform QA because beam data can vary from machine to machine, even for the same model and vendor, as described by AAPM MMPG 5.a, TG 51, 100, and 106 [1–3, 20].

## Conclusions

The field size for a 6-MV FFF radiation beam from the Halcyon system was determined using the simple SCF method. This method covers all field sizes, including small field sizes. The coefficients for the fitting and the field sizes between the RD and the MD were in good accord. This method can be used as a repeated countercheck for users using the same LINAC model.

## Data Availability

The datasets used and/or analyzed during the current study are available from the corresponding author on reasonable request.

## Abbreviations

QA: Quality assurance
IMRT: Intensity modulated radiation therapy
VMAT: Volumetric modulated arc therapy
SCF: Sigmoidal curve fitting
RD: Reference data
MD: Measured data
AAR: Average agreement ratio
IR: Introductory region
GR: Growing region
PR: Plateau region
SP: Singular point
IP: Inflection point
DFS: Determined field size
